# Geometric Triangular Chiral Hexagon Crystal-Like Complexes Organization in Pathological Tissues Biological Collision Order

**DOI:** 10.1371/journal.pone.0001282

**Published:** 2007-12-12

**Authors:** Jairo A. Díaz, Natalia A. Jaramillo, Mauricio F. Murillo

**Affiliations:** Laboratory of Pathology, Department of Pathology, Clinic Health Social Entity Policarpa Salavarrieta, University Cooperativa of Colombia, Medicine School, Villavicencio, Meta, Colombia; University of Freiburg, Germany

## Abstract

The present study describes and documents self-assembly of geometric triangular chiral hexagon crystal like complex organizations (GTCHC) in human pathological tissues.The authors have found this architectural geometric expression at macroscopic and microscopic levels mainly in cancer processes. This study is based essentially on macroscopic and histopathologic analyses of 3000 surgical specimens: 2600 inflammatory lesions and 400 malignant tumours. Geometric complexes identified photographically at macroscopic level were located in the gross surgical specimen, and these areas were carefully dissected. Samples were taken to carry out histologic analysis. Based on the hypothesis of a collision genesis mechanism and because it is difficult to carry out an appropriate methodological observation in biological systems, the authors designed a model base on other dynamic systems to obtain indirect information in which a strong white flash wave light discharge, generated by an electronic device, hits over the lines of electrical conductance structured in helicoidal pattern. In their experimental model, the authors were able to reproduce and to predict polarity, chirality, helicoid geometry, triangular and hexagonal clusters through electromagnetic sequential collisions. They determined that similar events among constituents of extracelular matrix which drive and produce piezoelectric activity are responsible for the genesis of GTCHC complexes in pathological tissues. This research suggests that molecular crystals represented by triangular chiral hexagons derived from a collision-attraction event against collagen type I fibrils emerge at microscopic and macroscopic scales presenting a lateral assembly of each side of hypertrophy helicoid fibers, that represent energy flow in cooperative hierarchically chiral electromagnetic interaction in pathological tissues and arises as a geometry of the equilibrium in perturbed biological systems. Further interdisciplinary studies must be carried out to reproduce, manipulate and amplify their activity and probably use them as a base to develop new therapeutic strategies in cancer.

## Introduction

The present study describes and documents self-assembly of geometric triangular chiral hexagon crystal like complexes organizations (GTCHC) in human pathological tissues. This architectural geometric expression has been found at macroscopic and microscopic levels mainly in cancer processes. In the literature, geometric triangular tissue morphological patterns have been documented at different levels:

At microscopic level: Triangular morphology of neoplastic cells in vitro was seen in rat cell sarcoma culture lines in relation with malignancy. [1]

At macroscopic level: Videoscopic triangular pattern images from the viewpoint of three-dimensional configuration of isolated crypts constitute an important factor in the gross assembly of colorectal tumours, [2] principally with a type IIIL. [3]

Triangular microcalcifications configuration [4] and stellate mammography images (triangular aggregation) are closely linked to malignancy [5].

**Figure 1:**
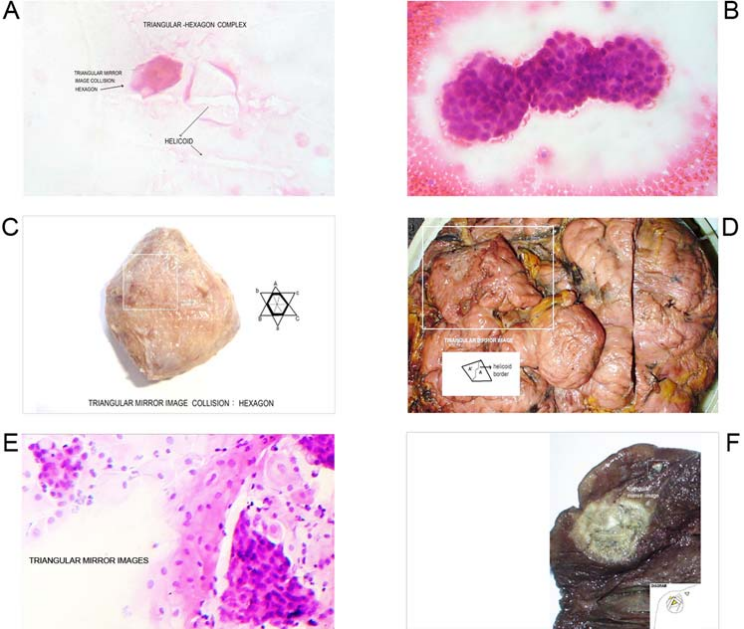
Geometric Triangular chiral hexagon cristal like complexes. Panel A. GTCHC complexes found in malignant peritoneal effusion, observe the helical pattern associate. Panel B. Breast malignant cells hexagonal clusters. Panel C. Colon polypoid juvenile tumor. Icosahedron pattern structuring by hexagon geometric stuctucture. Panel D. Macroscopic GTCHC complex in leiomyosarcoma. Panel E. Smear show triangular mirror images of atipical endocervix cells, that biopsy demostrated in situ adenocarcinoma. Panel F. Macroscopic GTCHC complex in lung cancer.

Despite these observations there is not corresponding references in human structural pathology about geometric self- assembly of triangular chiral hexagon complex organizations in pathologic tissues principally in malignant lessions, and it is not unknown how these images and their biological meaning are configurate. In this study the hexagonal morphology is evoked not from a simple six sides geometrical structure, but based on our images the authors consider it like a perfect organizational structure formatted from three organized triangular units with their respective mirror images . (Figure 1A, 1B, 1C, 1D, 1F)

A methodological analysis of 3000 images has allowed the authors to discover that the complexes are not uncommon or random findings on the contrary they appear as self –assembled structures that fit cohesively inside a morphological pattern, which assembles in space-time intervals, and consequently the authors believe they have a biological meaning .

By analyzing these complexes, repetitive morphological patterns that are inherent objectively to the configuration of each formation, and that belong to the field of physics are identified. The authors mention concepts like polarization, chirality, helicoidal pattern and geometric expression as a convergent characteristic of the images. Moreover, the authors in this study propose that the complex GTCHC identified in pathological tissues emerges from sequential collision processes that generate electromagnetic fields. Polarized photon collision of elementary particles like fermion produces chirality [6].

## Materials and Methods

### A) Observational support

The present research is based essentially on macroscopic and histopathologic analysis of 3000 surgical specimens: 2600 inflammatory lesions and 400 malignant tumours taken from the authors' patients received at the Public Health Clinic Carlos Hugo Estrada Pathology Service Laboratory in Villavicencio Meta during the last three years.

For the first time, the identification of GTCHC configurations was made on the center of a giant tumor of retroperitoneal localization (Figure 1D) under the premise of determining whether this strange formation was a casual discovery or if it represented a real biological fact. The authors decided to “look for this structures” in different pathological entities, to do so interesting specimens, were photographed, the pictures from different rotationary angles were analyzed, and the images were amplified two to three times.

Geometric complexes identified photographically at the macroscopic level were located in the gross surgical specimen; these areas were carefully dissected and samples were taken from the border lines, inside and outside of the GTCHC complexes, to carry out histological analysis and make a comparison with the macroscopic findings. The authors also worked with peritoneal, pleural effusions of malignant tumors; when a pattern was detected, it was also documented. For histological observation, all the materials were stained with hematoxylin and eosin routine and Tricromico of Masson coloration. Malignant smears were stained with papanicolaou.

Geometric complexes were considered valid if they presented volumetric assembling, if it was possible to find patterns at different levels of the surgical specimen, or if they appeared in more than four histology sections.

It is necessary to consider that GTCHC complexes do not emerge alone; rather, it is necessary to look for them. The search of the authors is similar to that of an archeologist, it is necessary to look carefully amid necrosis, hemorrhages and cystic changes of the tissue to conserve the original structure. When areas were cleaned a documentation process was developed by a photographic session, and it was here that the expression of an impressive geometric order emerged.

### (B) Experimental design

It is difficult to carry out an appropriate methodological observation for collision processes when studing biological systems. But one can obtain indirect information through models in other dynamic systems. What the authors were looking for mainly was to determine if one can reproduce and predict components of polarity, chirality, helicoid patterns, and geometric expressions through electromagnetic sequential collisions.

#### Collision event

Light and magnetism are reflections of the same substance. Light is an electromagnetic disturbance that propagates through a field; chirality, magnetism, and light are intimately correlated [7].

Under the hypothesis of a collision mechanism, the authors designed a model in which a strong white flash wave light discharge, generated by an electronic device, hits over lines of electrical conductance structured in helicoidal patterns, generating an electromagnetic field.

The objective of this experiment was to determine if the authors were able to reproduce polarization and chirality patterns, or triangular hexagonal geometrical configurations as a result of collision of two interdependent fields: in this case, light and electromagnetism.

For such effect, the authors use an electronic flash device attached to a Sony camera model DSC-S600. Strong discharges of light were made over electric conduction lines (150 volts) of helical pattern in time intervals of 3 to 4 minutes during cicles of 60 minutes from 3 to 4 m distance in an atmospheric environment and a low temperature of 4°C . To avoid lens flare, the experiment was performed in complete darkness. There were photographic sessions of 1 h during a 9-day time interval.

#### Statistical analysis

References between the two groups inflammatory benign and tumors malignant conditions where TGCHC complexes appear were determined using the Student test.

## Results

### Observational results

The authors detected GTCHC complexes at macroscopic and microscopic levels in human pathologic tissues.

**Figure 2 pone-0001282-g002:**
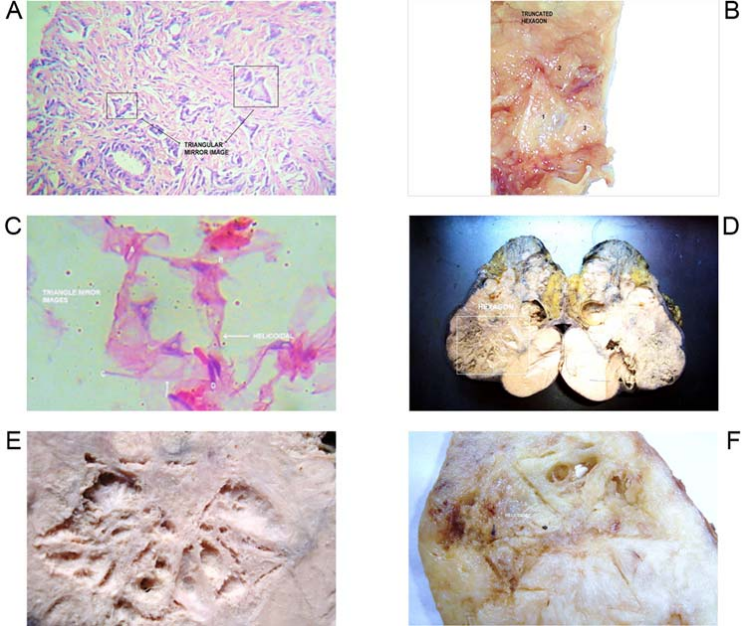
Geometric Triangular chiral hexagon cristal like complexes assembled from helicoid pattern. Panel A. Microscopic GTCHC complex in prostate adenocarcinoma. Panel B. Macroscopic GTCHC complex in liposarcoma. Panel C. Microscopic GTCHC complex in lipoma .Helicoid border. Panel D. Macroscopic GTCHC complex in breast cancer. Observe how the straight lines are assembled from helicoid border pattern. Panel E. Full view of GTCHC complex, this is the same as panel D after demarcating and cleaning the area. It is possible equally to identify “hole” microcavities in their surface. Panel F. Macroscopic GTCHC complex in filloid breast tumor with helicoid border pattern. We appreciate the initial formation of hole microcavities concluding in changes of cystic degeneration at the interior of the geometric complex.

**Figure 3 pone-0001282-g003:**
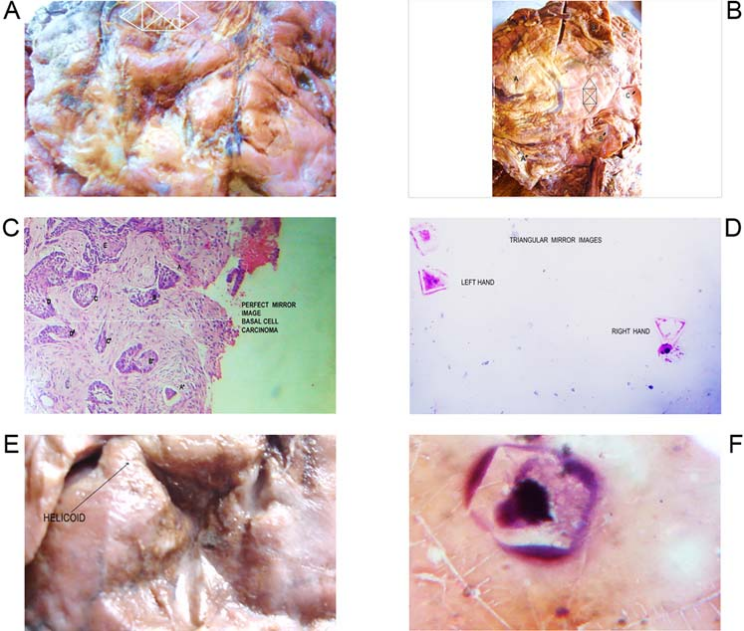
Geometric Triangular chira lhexagon cristal like complexes. Panel A. Macroscopic GTCHC complex in fibrosarcoma. Panel B. Macroscopic GTCHC complex in liposarcoma that induces surprisingly clear tissue mirror images around it: ABC––A'B'C'. Panel C. Microscopic GTCHC complex in basal cell carcinoma that induces surprisingly close tissue mirror images around it. ABCDE––A'B'C'D'E'. Panel D. Microscopic GTCHC in malignant peritoneal effusion. Panel E. Macroscopic GTCHC complex in malignant solid ovary tumor. Panel F. Microscopic fractional crystal hexagon from a cluster of malignant cells in peritoneal malignant effusion.

**Figure 4 pone-0001282-g004:**
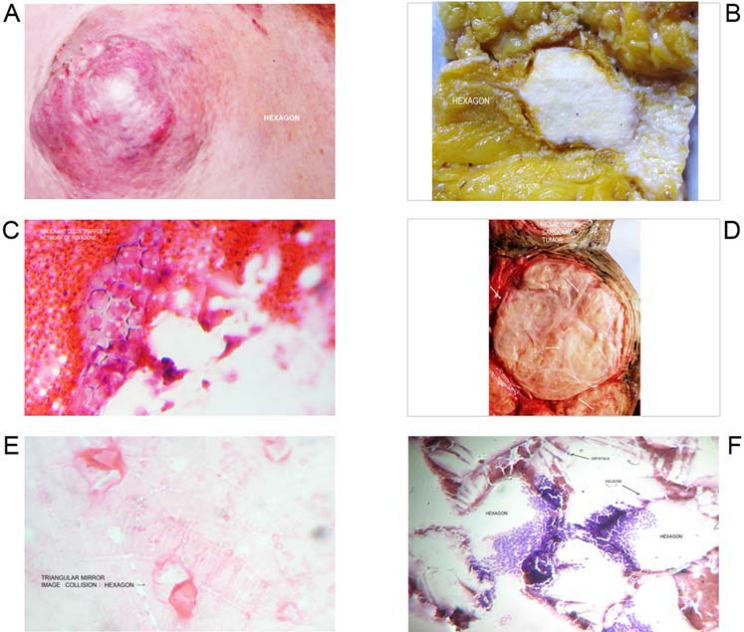
Geometric Triangular chiral hexagon cristal like complexes. Panel A. Hexagon pattern of breast cancer. Panel B. Breast cancer tissue surface cut hexagonal pattern. Panel C. Malignant breast cells trapped by a network of hexagons. Panel D. Hexagon pattern of fibrosarcoma nodule. Panel E. GTCHC complexes obtained from peritoneal malignant effusion. Panel F. Microscopic hexagonal pattern .Papilar thyroid tumor

From 2600 random inflammatory–immunologic processes analyzed, the authors identified images of triangular chiral counterpart tissue configuration in 312 cases (12%). From 400 cancer processes analyzed, geometric complexes were identified in 216 cases (54%). These percentages show identification of highly ordered structures in more than 50% of the analyzed malignant tumor tissues statistically significant (P = 0.00001) (Table 1). The organizations were observed with more frequency under tumor conditions (Figures 1–8) however also under benign condition (Figures 7D,7E ,7F, ). Sarcoma lesions of greater size were those that evidence more clear complex GTCHC (Figures 1D, 2B, 2F, 3A, 3B, 4D).

**Figure 5 pone-0001282-g005:**
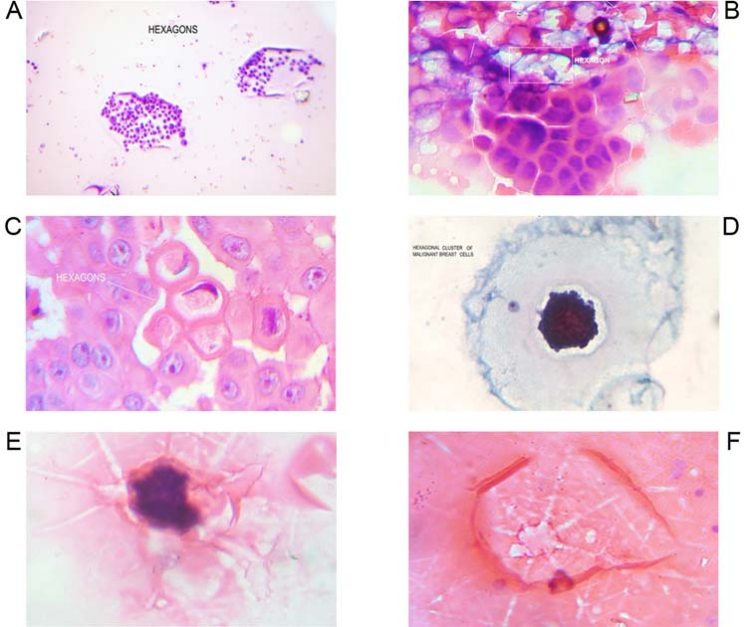
Morphodynamic sequence of malignant cells trapped at the interior of hexagonal cristal structure. GTCHC complex. Panel A–B. Malignant cells trapped inside crystal hexagons. Observe authofagic vacuolization. Panel C. Disintegrated malignant cells inside hexagons in sweat carcinoma with apoptosis changes. Panel D–E–F. Disintegrated malignant cells trapped inside crystal hexagons

**Figure 6 pone-0001282-g006:**
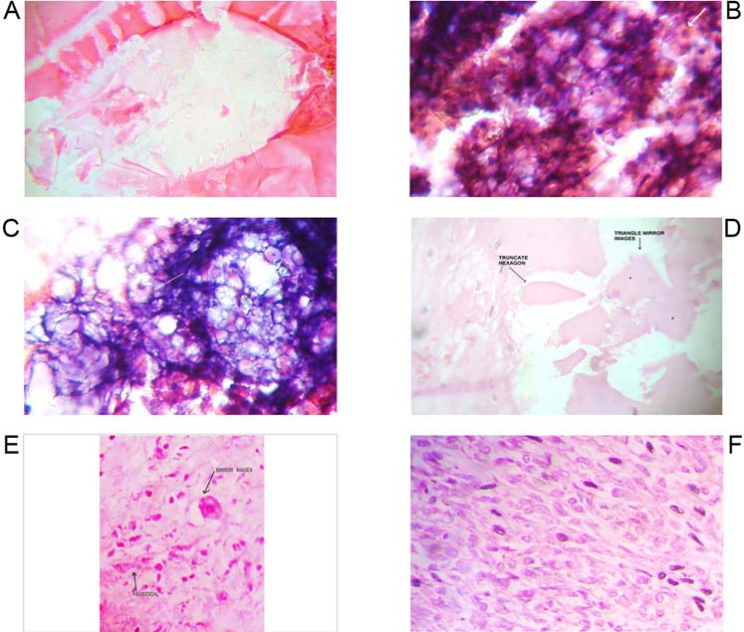
Final evolution step of GTCHC complexes. Panel A–B–C–D. Final step of massive malignant cell disintegration by hexagonal crystal complexes that remember carbon fullerene structure. Panel E. Histopathology findings inside macroscopic GTCHC: Low cellular population. Apoptosis, helicoidal hypertrophy of individual collagen type I bundles, and mirror images. Panel F. High cellular population, elevated mitosis typical characteristics of malignant behavior outside and near GTCHC complexes.

**Figure 7 pone-0001282-g007:**
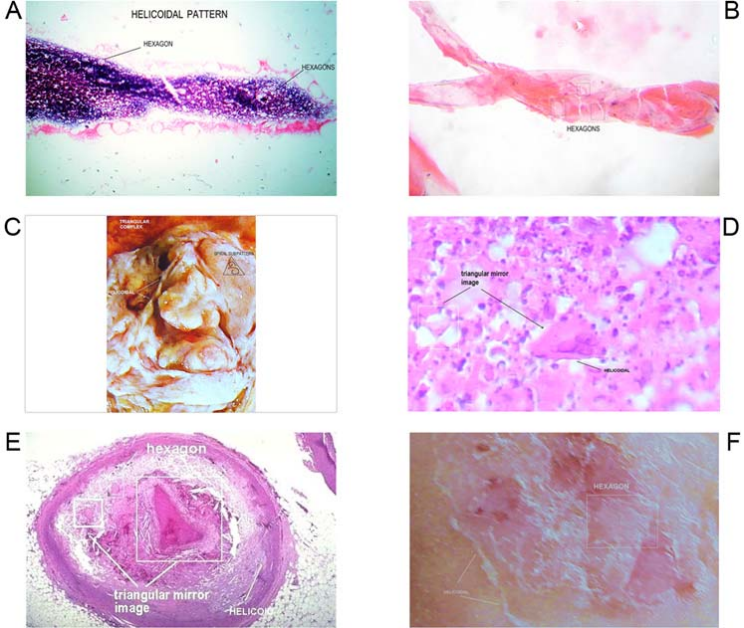
Collagen type I helicoid hypertrophy in GTCHC complexes. Panel A–B. Microscopic helicoid hypertrophy of individual collagen type I bundle, observed in malignant peritoneal effusion. Panel C. Macroscopic Triangular complex organization observed in breast cancer associate with helicoid hypertrophy of individual collagen type I bundle and spiral sub patterns. Panel D. GTCHC complex in benign lesion. Cells with herpes trapped inside triangular crystal structure with their chiral counterpart. Panel E. GTCHC complex in atherosclerosis. Panel F. GTCHC complex in superficial mycosis

**Figure 8:**
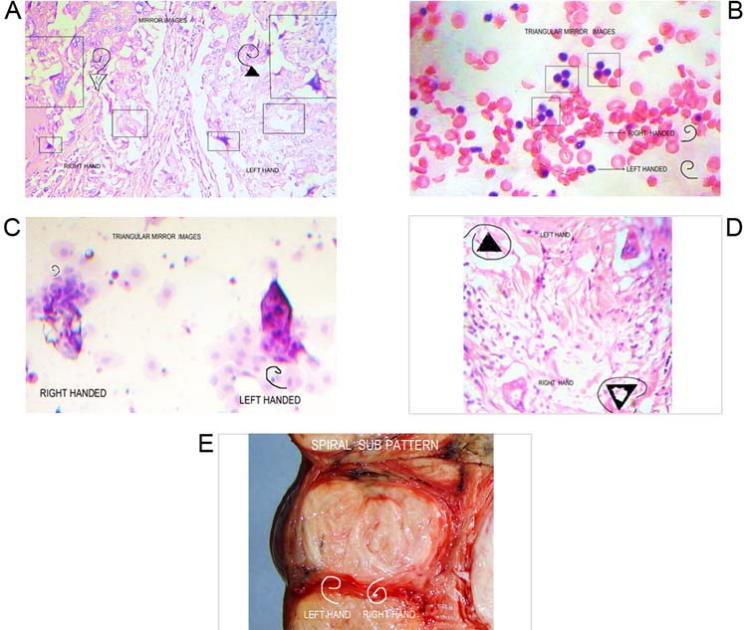
Spiral left handed , right handed sub patterns of GTCHC Complexes. Panel A. Microscopic view of breast ductal infiltrating adenocarcinoma. Observe how the images are plenty of mirror images and spiral sub patterns associated to GTCHC complex. Hypertrophic helicoid collagen fiber divides in defined left hand and right hand this specific zone of the tumor. Panel B. Smear from carcinomatosis abdominal ascitis .observe triangular mirror assembly of lymphocytes amazingly linked to spiral sub pattern of red Cells. Panel C. Smear from cervical displasia: Triangular mirror images with spiral sub pattern observe opposite dark cromatine condensation, white crystalization substrates of the geometries. Panel D. Prostate adenocarcinoma microscopic view. Opposite dark nuclear condensation, white authofagic vacuoles of triangular units. Panel E. Leiomiosarcoma macroscopic view. Nodules with spiral sub patterns.

**Table 1:** GTCHC complexes distribution in pathological tissues.

Cases	No	GTCHC	%
Inflammatory Benign conditions	2600	312	12
Cancer	400	216	54

The histopathologic analysis of the tissue at the interior of the complex showed accented cellular decrease, helicoid hypertrophy of collagen type I fibers, the presence of mirror images in lateral position, and apoptosis changes (Figures 6E,7A, 7B ). The lineal borders of the macroscopic complexes GTCHC also showed a pattern of helical hypertrophy in most of the cases (Figure 1D, 2C, 2F, 3E, 7C, 7F), additionally authors were able to discern two diferent morphological subtrates, dark condensation, or authofagic vacuoles depending on magnetic spiral sub patterns direction of the tringular complex and their counterpart (Figure 7C, 8 A, 8B, 8C, 8D, 8E) providing the fundamental proof that complexes are real. High cellular activity and frequent mitosis were the typical characteristics of malignancy outside of the complex GTCHC (Figure 6F) and were shown to have a completely opposite biological behavior. Cytological analysis of malignant peritoneal or pleural effusions evidenced the presence of crystals with triangular or hexagonal geometric composition (Figures 1A, 3F, 5A, 5B, 5E, 6A). These crystals are visible clearly in smears but difficult to observe in the cellular blocks of paraffin. From this the authors deduce that probably formaldehyde or the high temperature of the paraffin in the moment of inclusion of the material makes the crystals in histologic sections less evident.

### Experimental result

#### Collision event

In the experimental model, the collision of a strong flash from a white light against an electromagnetic field of electronic conduction lines produced the following morphodynamics sequential images.

**Figure 9:**
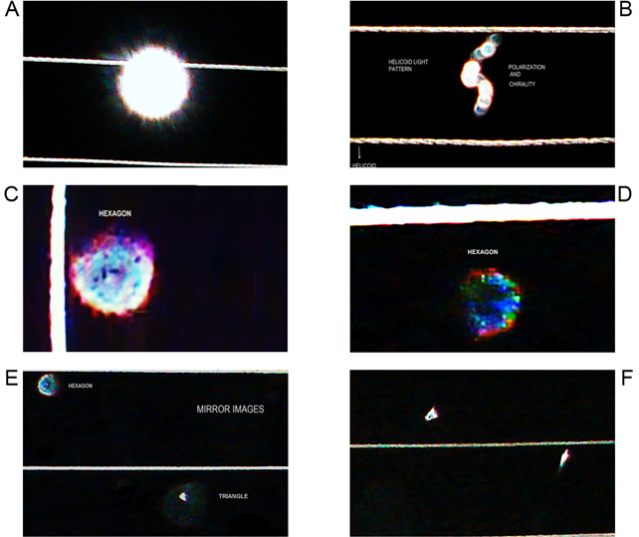
Morphodynamics sequential images of electro-optical dynamic system. Initial Momentum. Panel A. It illustrates a radian brigth that result from the collision interaction between the flash light and electromagnetic field. Panel B. In the interaction region result ejected particles of wave light that splitting into two components that take opposite directions in helicoid flow pattern with polarization and mirror image. Panel C–D. Each opossite light particles clusters begin to express hexagonal geometric configuration and “hole” microcavities in their surface.Higher magnification. Panel E–F. Triangular–hexagonal light pattern assembly in mirror images.

**Figure 10:**
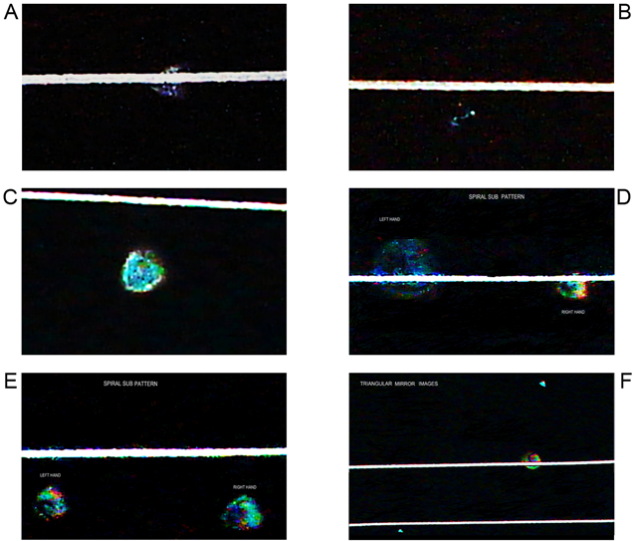
Morphodynamics sequential images of electro-optical dynamic system. Final Momentum. Panel A–B–C. Triangular light clusters experiences displacement inside the interaction region. Higher magnification. Panel D–E. Interleaving of sub patterns of indefinite light clusters and defined left handed and right handed spirals are structured in interaction area. Panel F. Finally triangular geometric expression twisted 360° at the boundaries of the system generatting mirror images (Chirality).

At the initial momentum of collision, the authors were able to appreciate a great white brightness (Figure 9A). In a dynamic process, in the interaction region result ejected particles of wave light that splitting into two components that take opposite directions in helicoid flow pattern with polarization and mirror images. The luminous energy trajectory is not in a straight line, but follows a helical pattern (Figure 9B). Triangular and hexagonal light patterns arises on interleaving of 15–20 sub patterns of indefinite light clusters and defined left handed and right handed spirals ( Figures 10D,10E), lateral to each side of the conduction lines, with “hole” microcavities in their surface (Figure 9C, 9D, 9E, 9F, 10A, 10B, 10C) within a space time intervals sequence discharges of 1 hour cicles. Images moved toward the boundaries of the perturbed system, diminishing their luminous intensity and appear distant. In this process, the authors observe a twisted of 360 degrees in invested position one from another (Figure 10 F).

## Discussion

Pathological tissues are an excellent means of analyzing chaos states and molecular disorder, and identifying order expression through geometry and mirror images in the core of this biological environment, it is a unique fact, and it is to be located in the chaos–order interface. All systems in which their particles are in continuous dynamic movement submit to laws of physics. This means that during the period space–time interval of life of any system, there exists the absolute potential to enter into a collision state at a given moment.

This logical premise is objective and real when one analyzes the structural pathological morphology from simple inflammatory processes such as the edema, the vasodilatation, and the migration of leukocytes, to malign proliferative processes where masses are generated and increase volumes, disorganizing extracellular matrix at extreme.

Collagen type I is the main protein of the extracellular matrix and has a crystal structure [8], and this characteristic allows collagen-type fibrils to arrange into a parallel order by regulatory functions of fibroblast cells, being this way in charge of the dielectric properties of natural tissues. The piezoelectric effect is a phenomenon resulting from a coupling between the electrical and the mechanical properties of a material. When mechanical stress is applied to a piezoelectric material, electric potential will be produced. Likewise, when an electric potential is applied to the material, a mechanical change will occur [9]. Piezoelectric crystals involve a nonuniform charge distribution within the cell unit of the crystal. When exposed to an electric field, this charge distribution shifts and the crystal changes its shape. The same polarization mechanism can cause a voltage to develop across the crystal in response to mechanical force; in pathological states of great disorder like cancer tissues, the extracellular matrix and most of the constituents of the system experience an enormous interaction impact that is expressed in piezoelectric activity. The collagen type I has a molecular structure based on quasihexagons [10].

Based on the observed images, the authors believe that stimulated collagen type I fiber at the moment of collision results in hypertrophy of helicoid patterns and their molecular crystal components are fractioned, being positioned to each side of the bundle fibril axis generating polarization and chirality (Figure 1A, 3D 3F, 4E, 4F, 6A, 6D, 7A, 7B). Straight lines of GTCHC complex associate directly with the helicoid pattern of the lateral borders. The authors believe that this represents the flow of energy through the fiber (Figure 7C).

There are many examples of such orientation induction on elongated supra/macromolecular structures in the literature using electric fields [11] or magnetic fields [12]. Chirality seems to be intimately associated with the growth and the stability of self-assembled fibrillar networks, and additionally molecular chirality is expressed in a scale of nanometers or micrometers, and helps twisted or coiled structures. There exists a specific link between helicoid patterns and chirality [13].

In their experimental model, the authors were able to reproduce and predict polarity, chirality, helicoid geometry, triangular and hexagonal clusters, starting from the impact of a wave of light on an electromagnetic field generated from an electric conduction line (Figures 9 and 10). A similar event of collision this time among constituents of extracellular matrix that drives and produces piezoelectric activity are responsible for the genesis of GTCHC complexes in pathological tissues.

In a collision state, electromagnetic field generates electrons, and ions split-up and take opposite trajectories and travel to the boundaries of the system where the particles twisted 360° one of another, and appear as mirror images. In physics this is determined as chirality, and chirality is a property of most of the biomolecules. This means the biomolecules are able to produce mirror images. This is one of the vital characteristics that are seen in pathological tissues, polarization from its side induces chirality, and polarization collision of elementary particles like fermion produces chirality [6]. Polarization formation in organisms shows similarity to an effect reported in crystals [14]. These data are coherent with the morphological findings. An experimental design was realized at low-temperature atmospheric conditions in this environment with water molecules of oxygen and hydrogen structured as ice crystals in hexagonal shapes. In this medium, when light dielectric discharges are in collision with this type of crystals, refraction deviation appears and generates clear hexagonal geometry (figure 9C, 9D). Spatio temporal patterns in dielectric discharge in air at atmospheric pressure have shown a rich variety, including traveling hexagon, traveling square, quasicrystals static hexagon, and stripe geometry [15]. By this way it is proved that polarization, chirality, helicoid patterns, and geometric expression can be in intimate relation with collision events and the electromagnetic field that it generates. The laws of the physics are amazingly universal as they serve from common denominators to dissimilar systems. And when geometric images obtained in the experimental pattern of collision are compared, they are amazingly similar to the complex GTCHC identified in pathological tissues, clearly in the point of collision of two systems geometry of equilibrium is generated.

The essential base of this geometry is the triangulation and the generation of mirror images that conclude in hexagons. Here hexagons are not a simple six-side structure; they are a complete state, net balance structured in the base of three triangular units, with their respective mirror images as a state of molecular order. The authors state that the crystal structure of water, liver tissue, alveolar walls, and the structuring of the fullerene particle of carbon is based on hexagonal geometric patterns that represent definitively a structural reorganization. Triangulation is then a consequence of successive and sequential impacts inside a density of particles in movement similar to the angular trajectory of a billiard ball that rotates freely in a flat surface and collides with obstacles.

The authors consider definitively that complex GTCHC is a mechanism of structural reorganization in perturbed biological systems modulated through geometry and represent orderly spatio-temporal distribution of positive and negative electric discharges inside the system. The authors document macroscopic and microscopic images that demonstrate how clear mirror images form in proximity to the complex GTCHC, and it seems to originate from the geometrical basis of this complex (Figure 3B, 3C, 8A) the same one that indicates that these structures would have magnetic properties, links between magnetism, and chirality exist [7].

Inside their crystal structure, GTCHC complexes can trapped a virus (Figure 7D) an anomalous lipoprotein (Figure 7E), or a malignant cell, and they disintegrate it. In malignant lesions, this morphodynamic sequence can tracked microscopically the cells are somehow caught in a complex (Figure 4C), or disintegrated at the interior of the crystal structure (Figure 5A, 5B, 5E, 5F, 6A, 6B). The complex GTCHC multiplies inside the perturbed system, similar to fullerene carbon structure (Figure 6B, 6C).

### How did the authors interpret these images?

All biomolecules under normal conditions, that is to say in stable balance, have their respective mirror images; in biological disorder state this balance gets lost and their chiral counterpart disappears. This loss of mirror images imbalances the system to more chaos and bigger imbalance of structural polarity. This is what happens in malignant tumors that have lost polarity and have architectural disorder. GTCHC complex brings polarity to the disorder system and is represented in triangular mirror images that conform to hexagons. The research' images demonstrate a narrow relationship, at least morphologically, between the microscopic and macroscopic level. The link between macroscopic microscopic phenomena and molecular chirality proves that crystals are necessary for the direct assignment of absolute configuration of chiral molecules [16]. The authors believe that the complexes GTCHC have a biological meaning in conformity with the microscopic and macroscopic finds. In tumour injuries, microscopically focal authofagic vacuolization and apoptotic changes are observed without elements of inflammatory or necrotic visible partners to these structures (Figure 5B, 5C). The interrelation between these changes represent probably the cystic formations that are visualized at macroscopic level inside these complexes (Figure 2E, 2F), which would inhibit the pathological progression of the tumor and its expansion. Geometries would be a transcisional dynamic step toward the known process of malignant tissues cystic degeneration

Under deductive analysis it is possible to affirm that in malignant tumors left handed and right handed order exists. Authors discern two oppossite diferent types of morphological subtrates, dark cromatine condensation, proliferative status and authofagic vacuoles apoptosis degenerative cystic changes, depending on magnetic spiral sub patterns direction of the tringular basic units of the GTCHC complexes. (Figures 7C, 8A, 8B, 8C, 8D).

This research suggests that molecular crystals represented by triangular chiral hexagons derived from collision events against collagen type I fibrils emerge at microscopic and macroscopic scales in lateral assembly of each side of hypertrophy of helicoid fibers that represent flow energy in cooperative hierarchically chiral electromagnetic interaction in pathological tissues, and arises as a geometry of the equilibrium in perturbed biological systems .

The authors recommend that further interdisciplinary studies be carried out to investigate these geometric complexes to know them better and in the future, to be able to reproduce, manipulate, and amplify their activity and maybe use them as a base to develop new therapeutic strategies in cancer. It is thinkable reorganize a perturbed system through the spatio-temporal assemby of mirror image molecules.
